# Exploring the mechanism of aidi injection for lung cancer by network pharmacology approach and molecular docking validation

**DOI:** 10.1042/BSR20204062

**Published:** 2021-02-12

**Authors:** Zhenjie Zhuang, Tong Lin, Lixia Luo, Weixin Zhou, Junmao Wen, Haifu Huang, Zhanhua Liu, Lizhu Lin

**Affiliations:** 1 Guangzhou University of Chinese Medicine, Guangzhou, China; 2 Department of Oncology, Shenzhen Hospital of Guangzhou University of Chinese Medicine, Shenzhen, China; 3 Department of Oncology, The First Affiliated Hospital of Guangzhou University of Chinese Medicine, Guangzhou, China

**Keywords:** Aidi Injection, lung cancer, network pharmacology, pharmacological mechanisms

## Abstract

***Background*.** Aidi injection (ADI) is an effective Traditional Chinese medicine preparation widely used for lung cancer. However, the pharmacological mechanisms of ADI on lung cancer remain to be elucidated. ***Methods*.** A network pharmacology (NP)-based approach and the molecular docking validation were conducted to explore underlying mechanisms of ADI on lung cancer. The compounds and target genes were screened by Traditional Chinese Medicine Systems Pharmacology (TCMSP) database and Bioinformatics Analysis Tool for Molecular mechANism of Traditional Chinese Medicine (Batman-TCM) database. The STRING database was utilized for protein interaction network construction. The R package *clusterProfiler* was used for bioinformatics annotation of hub target genes. The gene expression analysis and survival analysis were performed based on The Cancer Genome Atlas (TCGA) database. The Autodock Vina was used for molecular docking validation. ***Results*.** A total of five key compounds with 324 putative target genes were screened out, and 14 hub target genes were identified for treating lung cancer. Six hub genes could influence the survival of non-small cell lung cancer (NSCLC) patients. Of these hub genes, the expression pattern of *EGFR, MYC, PIK3CA*, and *SMAD3* were significantly higher in the LUSC, while *PIK3CA* and *RELA* expressed lower in the LUAD group and LUSC group, respectively. These six hub genes had good docking affinity with the key compounds of ADI. Kyoto Encyclopedia of Genes and Genomes (KEGG) pathway analysis showed that ADI may exert therapeutic effects on lung cancer by regulating critical pathways including the thyroid hormone signaling pathway, MAPK signaling pathway, and PI3K-Akt signaling pathway. ***Conclusions***. The present study explored the potential pharmacological mechanisms of ADI on lung cancer, promoting the clinical application of ADI in treating lung cancer, and providing references for advanced researches.

## Introduction

Lung cancer is a dominating cause of cancer-related mortality among middle-aged and elderly people, leading to a total of 145849 deaths in 2017 [1]. According to its pathology, lung cancer comprises two major categories: non-small cell lung cancer (NSCLC) and small cell lung cancer. NSCLC makes up nearly 85% of lung cancer cases [2]. At present, standard therapies for NSCLC include conventional chemotherapy, radiation therapy, targeted therapy and immunotherapy, which are cost-effective and may increase the risk of adverse events [3–6]. Therefore, more reasonable treatment options with less cost and fewer treatment-related adverse events are needed for patients with lung cancer.

Traditional Chinese medicine (TCM), as one kind of complementary and alternative medicine, has been widely applied clinically for more than 2000 years in China, and TCM is becoming more frequently used all over the world nowadays [7]. In recent years, TCM preparations (TCMPs) have been proposed as one of the crucial options for the treatment of cancers [8]. Aidi injection (ADI) (Z52020236, China Food and Drug Administration), one of the multitarget anti-tumor Chinese patent medicines, is an adjuvant TCMP commonly used in the treatment of NSCLC in China [9]. ADI is derived from four Chinese ingredients including *Mylabrisphalerata pallas* (Banmao [BM]), *Astragalus membranceus (Fisch.) Bge.* (Huangqi [HQ]), *Panax ginseng C.A.Mey.* (Renshen [RS]), *Acanthopanax senticosus (Rupr. and Maxim.)*, and *Harms* (Ciwujia [CWJ]) [10]. These ingredients all meet the standards for drug use and development referring to Chinese Pharmacopoeia (version 2015) [11]. Modern pharmacological studies have shown that ADI possesses the ability to inhibit the proliferation of A549 cells, reduce hepatotoxicity and gastrointestinal toxicity, and improve immunity [12–17]. Besides, several meta-analyses report that ADI combined with chemotherapy make great improvements in clinical efficacy and quality of life (QoL) in patients with NSCLC and also reduce adverse events induced by chemotherapy [18–21]. However, the underlying mechanisms of ADI in treating NSCLC remain vague and warrant further investigation.

With the development of TCM modernization, network pharmacology (NP) has emerged as an advantageous method for TCM research, which could provide a new viewpoint at molecular level [22]. NP can generate complicated interaction networks based on target molecules, active compounds and biological functions, which meets the natural characteristics of TCM formula and helps to clarify their underlying mechanisms [23,24]. In recent years, researchers have employed NP methods to elucidate the active ingredients and mechanisms of TCM against various diseases [25]. In the present work, NP was developed to investigate the effective compounds and pharmacological mechanisms for ADI acting on NSCLC.

## Materials and methods

### Database establishment

The drug compound and target genes of four ingredients of ADI were gathered from the Traditional Chinese Medicine Systems Pharmacology database (TCMSP, http://lsp.nwu.edu.cn/tcmsp.php) [26] and Bioinformatics Analysis Tool for Molecular mechANism of Traditional Chinese Medicine (Batman-TCM, http://bionet.ncpsb.org/batman-tcm/index.php) [27]. TCMSP provides 499 Chinese herbs with 29384 ingredients, and their targets, related diseases, as well as chemical structural data etc. [26]. Under the guidance of TCMSP, oral bioavailability (OB) and drug-likeness (DL) of the compounds were set as greater than or equal to 30% and 0.18, respectively. We also utilized the UniProt database [28] to verify the target gene symbol from TCMSP. Batman-TCM provides a score for every target gene of the compound, and the value of the score is positively correlated with the reliability of the target gene [27]. In the present study, only the target genes with a score greater than the mean score of all target genes of ADI were included. Compounds and target genes from TCMSP and Batman-TCM were merged for constructing the drug compounds and target gene database.

### Identification of putative target genes for lung cancer

The putative target genes of lung cancer were obtained from DisGeNET (version 6.0), Online Mendelian Inheritance in Man (OMIM), and Therapeutic Target Database (TTD). DisGeNET contains multiple and integrative data of target genes and their related human diseases [29]. OMIM provides more than 15000 genes information together with the related diseases mainly based on published scientific literature [30]. TTD offers data of 34019 drugs and their corresponding target genes, as well as related diseases and pathways [31]. The putative target genes collected from three databases above were combined to construct the target gene database of lung cancer.

### Construction of protein–protein interaction network

The putative target genes of ADI and lung cancer were overlapped to identify the shared target genes for ADI to treat lung cancer. These common putative target genes were input into the search tool for the retrieval of interacting genes (STRING) 11.0 database (https://string-db.org/) [32] to construct the protein–protein interaction (PPI) network. To guarantee the robustness of outcomes, the screening threshold in the STRING database was set as interactions score ≥ 0.9. Next, the PPI networks were visualized and analyzed using Cytoscape (version 3.72) [33]. Degree, betweenness, closeness were three important indexes to describe a protein's topological importance in the network. In the PPI network, nodes met with all the following topology value criteria were considered as hub target genes in the network: (1) with the degree greater than double of the median degree; (2) with betweenness greater than the median betweenness; (3) with closeness greater than the median closeness.

### Bioinformatics annotations of hub target genes

A functional R package called *clusterProfiler* was utilized to perform the Gene Ontology (GO) and Kyoto Encyclopedia of Genes and Genomes (KEGG) pathway enrichment analysis for the hub target genes [34]. The GO analysis including molecular function (MF), biological process (BP), and cellular component (CC) was calculated. *ClusterProfiler* is a famous R package with dynamically updating data for KEGG and GO analyses. The screening threshold result was set as *P*-value ≤0.01. In addition, the false discovery rate (FDR) was used for the adjustment of *P*-value.

### Identification of clinical significance of hub genes

The Cancer Genome Atlas (TCGA) is a landmark cancer genomics program exhibited by the National Cancer Institute and the National Human Genome Research Institute in 2005. TCGA furnishes more than 20000 primary cancer and matched para-carcinoma samples of 33 cancer types. The gene expression matrix and their corresponding clinical information of lung cancer including NSCLC tissues (Lung Adenocarcinoma, LUAD: 535; Lung Squamous Cell Cancer, LUSC: 502) and 108 contrasted normal tissues (LUAD: 59; LUSC: 49) with complete clinical information derived from 1014 NSCLC patients were acquired via an R package called *TCGAbiolinks* [35]. The gene expression matrix was normalized by an R package called *DESeq2* [36]. Then the survival analysis was conducted to explore the impact of the hub genes on the survival of NSCLC patients. The log-rank test and single gene Cox proportional hazards regression were applied to calculate the *P*-value together with hazard ratio (HR) with a 95% confidence interval (CI) between the two groups. The result of hub genes with statistical significance in both Cox regression and log-rank test would be presented. R package *survminer* [37] was applied to plot the survivorship curve and compute the cutoff value of expression of the hub genes. With the hub genes that affect the survival of NSCLC patients ascertained, their expression pattern would be further identified. The Shapiro–Wilk test was used for the normality test of the hub gene expression data. If the expression data met normality, a *t* test would be applied to check the significant difference between the cancer group and para-cancerous group. Otherwise, a Wilcoxon's test would be applied instead. R package *ggplot2* [38] was applied to draw boxplots of the expression pattern of hub genes.

### Drugs–compounds–hub target genes–pathways network construction

With hub genes and KEGG pathways identified, the drugs–compounds–hub target genes–pathways network was constructed by Cytoscape (version 3.72). In this network, the degree value was applied for filtering major hub genes and key compounds. The compound nodes whose degree value ranking in the top five of all the nodes were considered as key compounds of ADI for treating lung cancer. In addition, hub genes with the top three degree value were considered major hub target genes for ADI to treat lung cancer.

### Validation of key compound–hub target gene interaction

**Figure 1:**
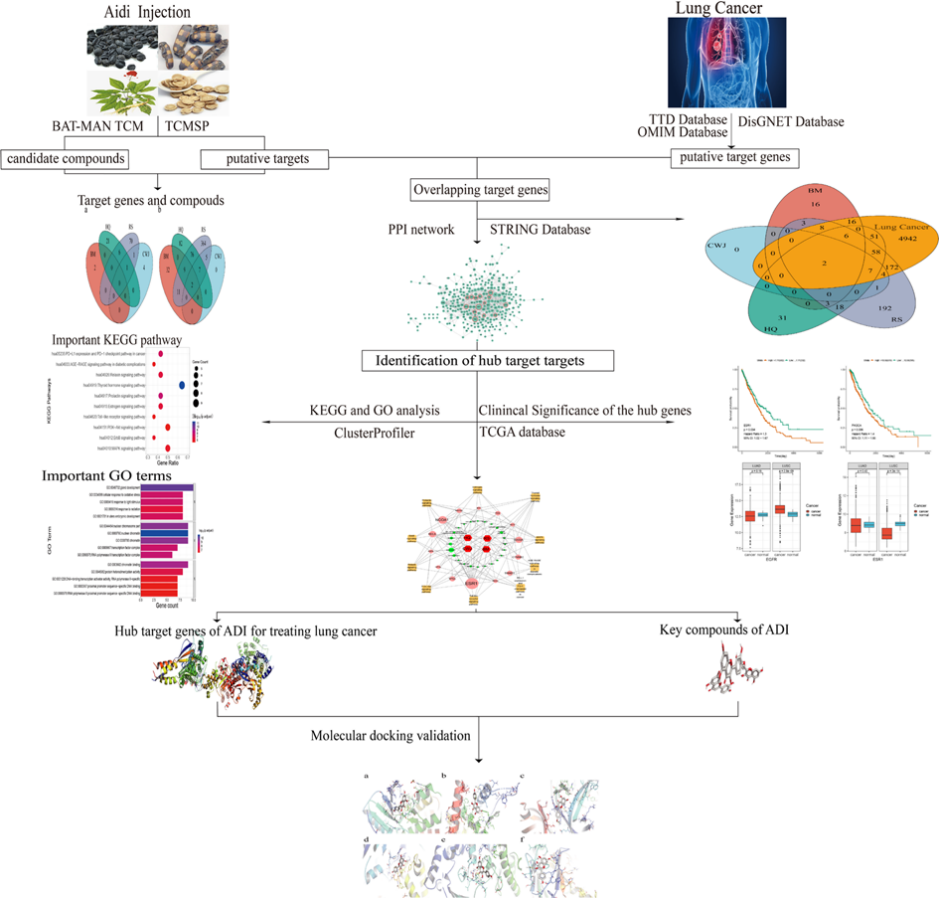
The flowchart of ADI in treating lung cancer based on NP

Furthermore, the molecular docking approach was utilized to validate the key compound–hub target gene association. The 3D structures of proteins expressed by the hub target genes with a significant impact on NSCLC patients’ overall survival were obtained from the RCSB PDB database (https://www.rcsb.org/). In addition, the 3D structures of the key compounds of ADI were obtained from the PubChem database (https://pubchem.ncbi.nlm.nih.gov/) as *SDF* format and converted into *PDB* format via Pymol software (version 2.2). Pymol software [39] was also used to remove the hydrone and the additional ligand of the hub target proteins. The AutoDockTools (version 1.5.6) [40] was applied for adding hydrogen atoms, merging nonpolar hydrogen atoms, computing the charge number of the protein, and detecting the docking site. Besides, the AutoDockTools was applied to set the key compounds as ligands and explore the structure torsion and root of ligands. Subsequently, both formats of all the hub target protein and key compounds were converted into *pdbqt* format. AutoDock Vina [41] was used to perform molecular docking between the hub target proteins and key compounds of ADI. The conformations of the key compounds and target protein were visualized by Pymol software (version 2.2). The docking conformation that has docking affinity score −5.0 kcal/mol represents great binding interactions between the compound and its corresponding targets [42]. The flowchart regarding the research procedures of the present study was shown in Figure 1.

## Results

### Quantification of active compounds and putative target genes of ADI

**Figure 2:**
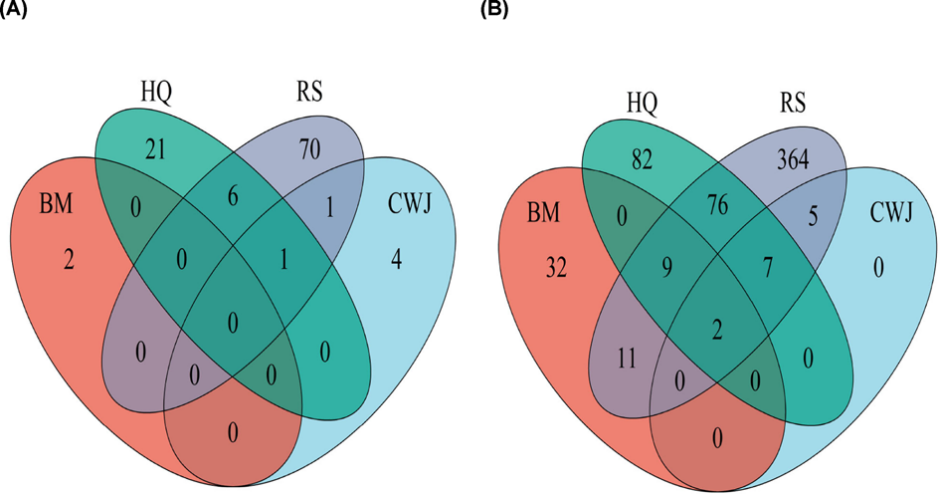
The Venn diagram of different compounds and target genes number distribution in ADI (**A**) The number distribution of different active compounds in ADI. The red oval represents the identified BM compounds. The green oval represents the identified HQ compounds. The gray and blue ovals represent the RS and CWJ compounds, respectively. (**B**) The number distribution of different putative target genes in ADI. The red and green ovals represent the identified BM and HQ targets, respectively. The gray oval represents the identified RS target genes. The blue oval represents the CWJ targets.

Based on both TCMSP and Batman-TCM databases, a total of 114 active compounds of the four Chinese medicines in ADI were identified. The total compound number of each Chinese medicine of ADI and the compound number they overlapped were shown in Figure 2A. The detailed information on drugs and compounds of ADI is shown in Appendix A (Supplementary material). In addition, a totally of 324 putative target genes of ADI were gathered. The amounts of putative target genes of BM, CWJ, HQ, and RS drugs were 54, 14, 176, and 474, respectively. More details are shown in Appendix B (Supplementary material). There were 2 putative target genes of the four Chinese medicines that overlapped and a significant target gene number overlapped between HQ and RS (76 putative targets), but lesser number overlapped between RS and BM (11 putative targets). The number of target genes shared by HQ, RS, and BM was 9, while 7 target genes were overlapped among HQ, RS, and CWJ (Figure 2B). The common compounds and common target genes shared between these four Chinese medicines suggested that they might exert synergistic therapeutic effects in the course of treatment.

### Quantification of putative target between lung cancer and ADI

**Figure 3:**
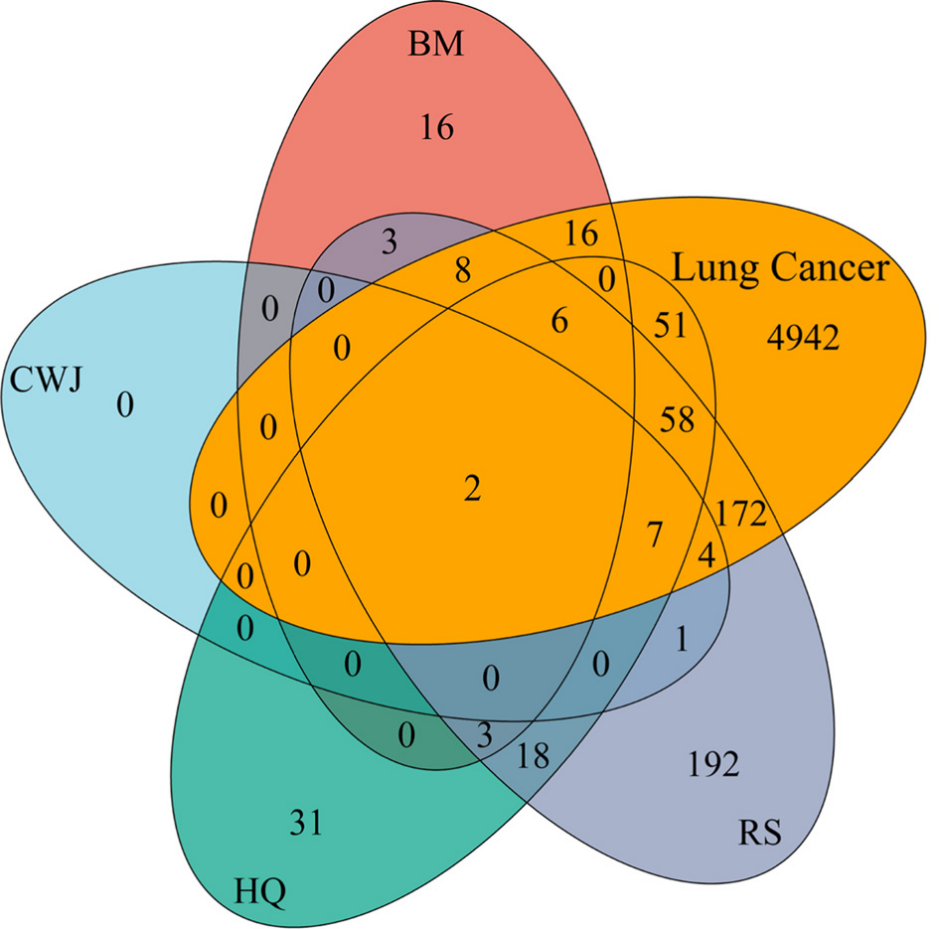
The Venn diagram of the number relationship between the putative target genes of ADI and lung cancer The red and green ovals represent the identified BM and HQ targets, respectively. The gray oval represents the identified RS target genes. The blue oval represents the CWJ targets. The yellow oval shows the total target genes of lung cancer.

The amount of putative target gene of lung cancer collected from DisGeNET database, OMIM database, and TTD database was 5209, 67, and 54, respectively. After eliminating the redundancy, a total of 5266 putative target genes were verified. The details are described in Appendix C (Supplementary material). By combining the data of putative target genes of ADI and lung cancer, 324 common target genes were identified in total. The details are shown in Appendix D (Supplementary material). These common putative targets may play important role in ADI treating lung cancer and was applied for the further analysis. The common putative target gene number between four Chinese medicines in ADI and lung cancer are shown in Figure 3.

### Analysis for the PPI network

**Figure 4:**
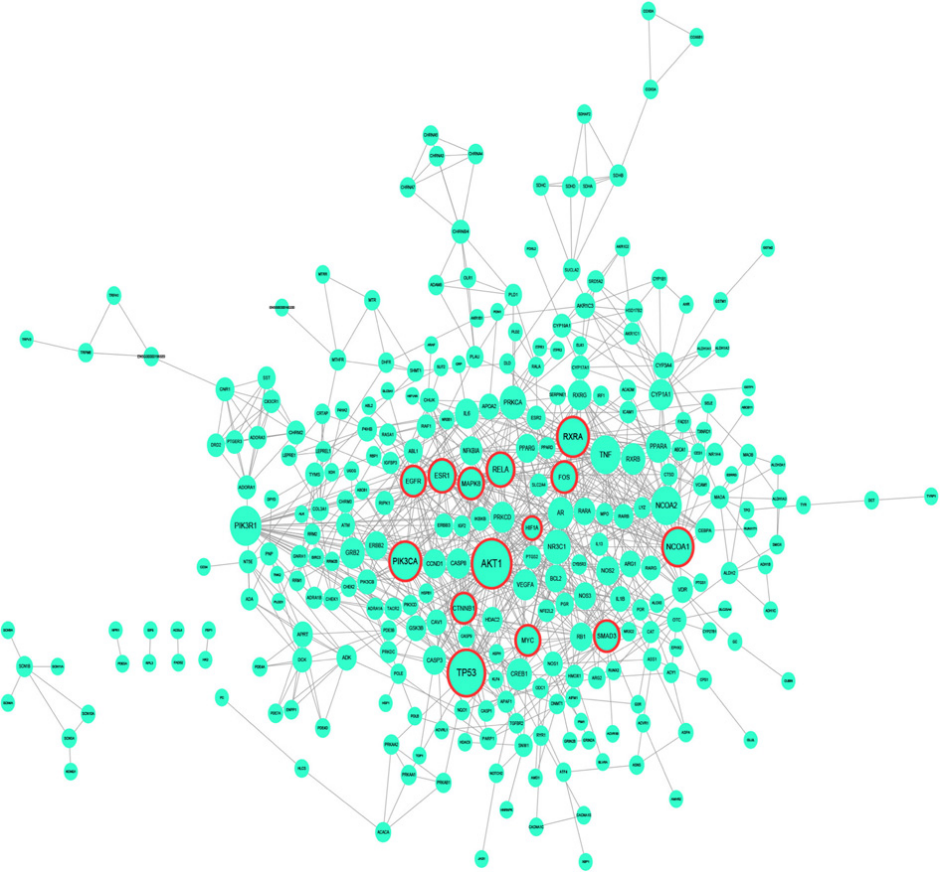
PPI interaction network of target genes shared between ADI and lung cancer Nodes with red border stand for hub nodes of the PPI network. The size of node is positive correlation with the degree of node in the network.

A total of 324 common putative target genes were input into STRING 11.0 to obtain the interactions of proteins and the Cytoscape 3.72 was utilized for the PPI network construction (Figure 4). As a result, the PPI network contained 283 nodes and 894 edges. Ultimately, 14 nodes proved to have degree value > 8 (double median value of degree), betweenness > 0.00198758 (above the median value), and closeness > 0.262279 (above the median value) were selected as hub nodes (the detailed information of 14 nodes in the PPI network is described in Table 1). Since these genes played an important role in the network, they were identified as the hub target genes for ADI to treat lung cancer.

**Table 1:** Information regarding hub gene of ADI for lung cancer

Hub gene	UniProt entry ID	Protein name	Degree	Closeness	Betweenness
*HIF1A*	F8W9L0	Hypoxia-inducible factor 1-α	12	0.35695187	0.03733165
*CTNNB1*	C9IZ65	Catenin β-1	20	0.34856397	0.02403861
*EGFR*	Q8NDU8	Epidermal growth factor	20	0.3463035	0.12291306
*MYC*	B3CJ73	V-myc myelocytomatosis viral oncogene homolog	20	0.35790885	0.02736214
*FOS*	G3V5N9	Proto-oncogene c-Fos	21	0.36081081	0.03624969
*MAPK8*	A0A3B3IRW7	Mitogen-activated protein kinase 8	21	0.34585492	0.02615858
*SMAD3*	H0YMY0	Mothers against decapentaplegic homolog 3	21	0.34407216	0.05659218
*ESR1*	C0LLI7	Estrogen receptor α	23	0.37238494	0.06912025
*RELA*	Q2TAM5	RELA protein	24	0.34362934	0.0234154
*NCOA1*	B5MCN7	Nuclear receptor coactivator 1	29	0.34362934	0.06069688
*PIK3CA*	E2I6G1	Phosphatidylinositol-4,5-bisphosphate 3-kinase catalytic subunit α isoform variant	30	0.3531746	0.04419187
*RXRA*	A0A3B3IS44	Retinoic acid receptor RXR-α	30	0.3718663	0.1161221
*TP53*	A0A0R9RRX7	Tumor protein p53	38	0.37083333	0.14175003
*AKT1*	X2CV47	AKT1m transcript variant 3	41	0.38472622	0.16982276

### Outcomes of the bioinformatics annotation

**Figure 5:**
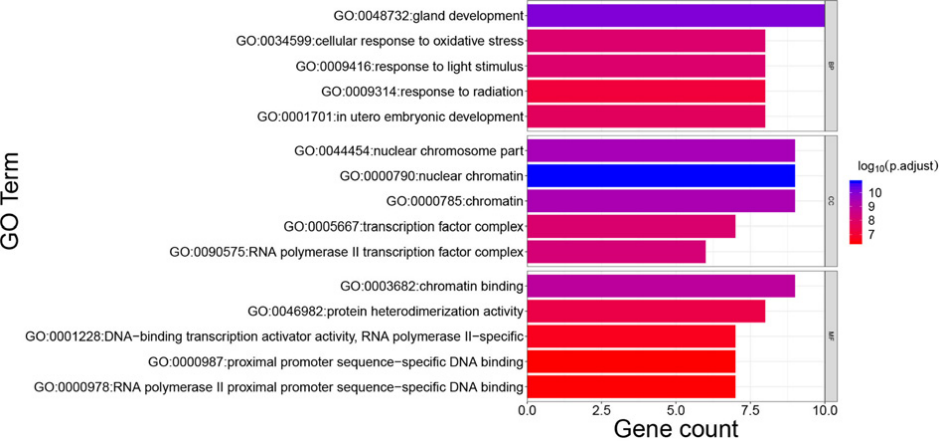
Main GO terms enriched by hub target genes The color of the terms turned from blue to red. The redder the bar is, the smaller the adjusted *P*-value is.

**Figure 6:**
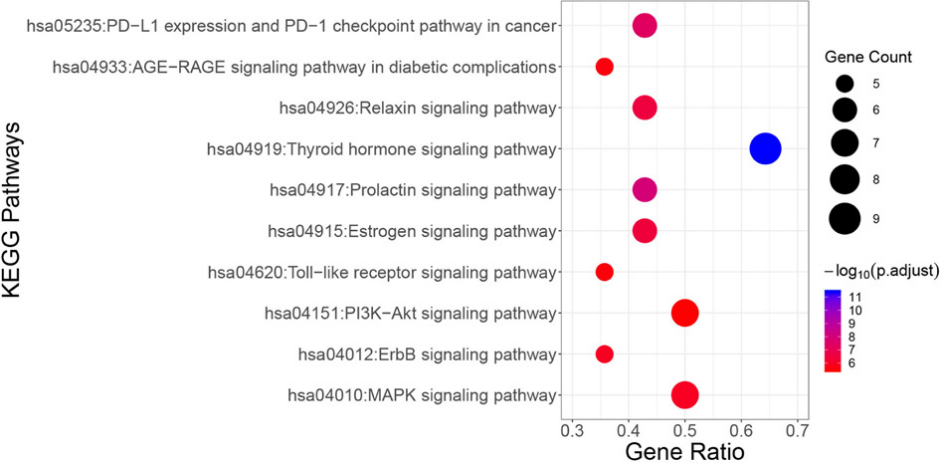
Main KEGG terms enriched by hub target genes The color of the terms turned from blue to red. The redder the bubble is, the smaller the adjusted *P*-value is. The enriched KEGG terms increase from small to large, and larger nodes indicate more enriched terms.

As for GO analysis, 1416 GO terms were defined, including 1334 of BP, 8 of CC, and 74 of MF enriched for these hub target genes. Top five terms of BP, MM, and CC with adjusted *P*-value were presented, respectively. (Figure 5). The major BP included gland development and cellular response to oxidative stress. The major CC included the nuclear chromosome part and the nuclear chromatin. Major MF included chromatin binding and protein heterodimerization activity. In addition, 153 KEGG pathways were recognized and the top 10 KEGG pathways with significantly adjusted *P*-value were presented (Figure 6). Results of the KEGG enrichment analysis indicated that the main pathways of the hub genes against lung cancer mainly focused on the thyroid hormone signaling pathway, PI3K-Akt signaling pathway, and MAPK signaling pathway. Therefore, ADI might treat lung cancer via GO terms and pathways above.

### Clinical significance of hub target genes in NSCLC

RNA-seq data of 1014 NSCLC patients were obtained. LUAD included 535 cancer samples and 59 normal para-carcinoma samples, while LUSC included 502 cancer samples and 49 normal para-carcinoma samples. With the TCGA data normalized, 14 hub genes met with the filter criterion were filtered from the PPI network and their clinical significance on NSCLC was identified by TCGA data. As a result of 14 hub genes, a total of 6 genes, *EGFR, ESR1, MYC, PIK3CA, SAMD3*, and *RELA* were proved to influence the survival of NSCLC patients.

**Figure 7:**
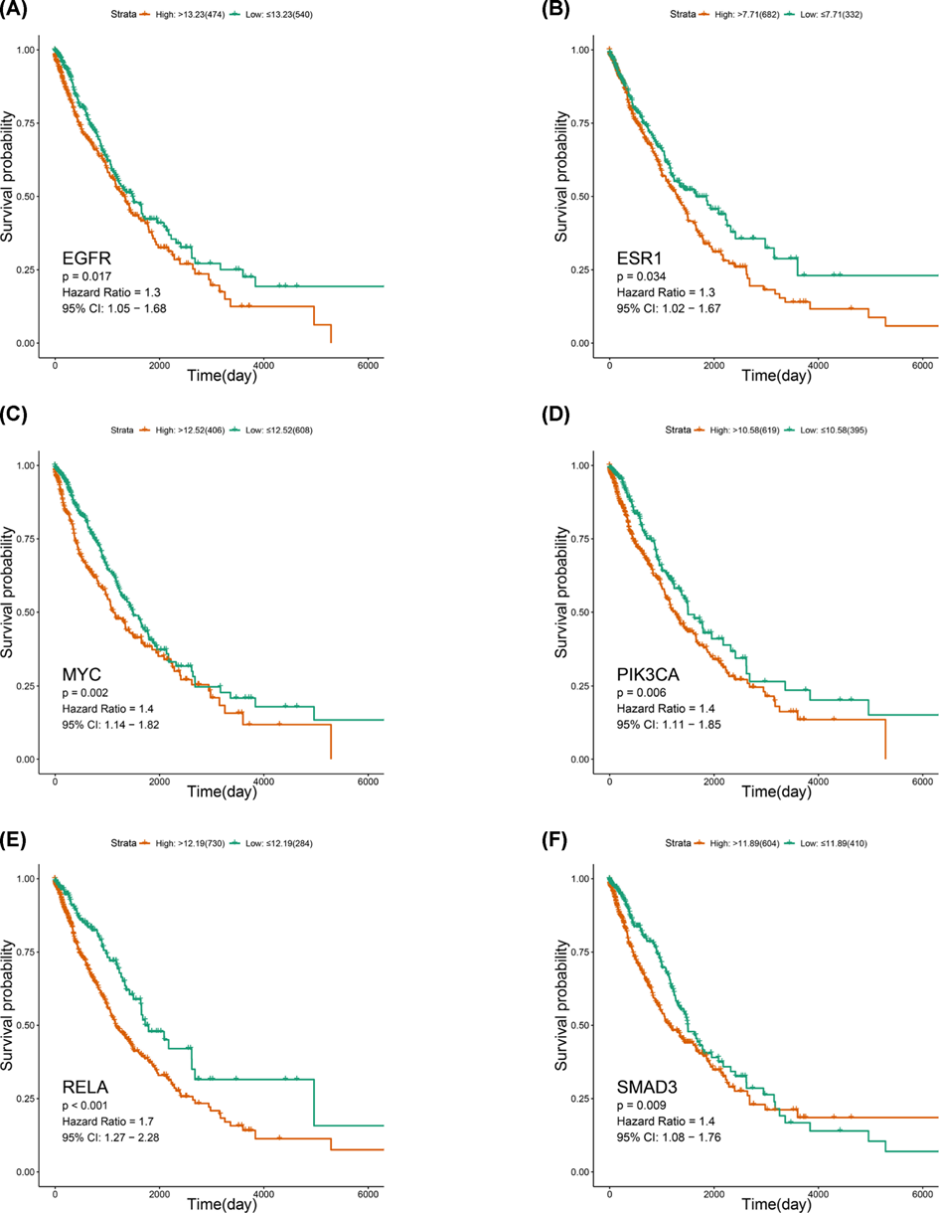
Clinical significance of *EGFR, ESR1, MYC, PIK3CA, RELA*, and *SMAD3* (**A**–**F**) Survival curves of *EGFR, ESR1, MYC, PIK3CA, RELA*, and *SMAD3* in NSCLC patients.

Higher level of *EGFR* expression was significantly associated with a poorer survival than compared with low expression in NSCLC patients (cutoff value of expression: 13.23; *P*-value: 0.017; HR: 1.3, 95% CI: 1.05–1.68), and this outcome resembled the *ESR1* gene (cutoff value of expression: 7.71; *P*-value: 0.023; HR:1.3, 95% CI: 1.02–1.67) (Figure 7A,B). Higher level of *MYC* expression also had significant association with a poorer survival (cutoff value of expression: 12.52; *P*-value: 0.002; HR: 1.4, 95% CI: 1.14–1.82), which was similar to *PIK3CA* (cutoff value of expression: 10.58; *P*-value: 0.006; HR: 1.4, 95% CI: 1.11–1.85), *SMAD3* (cutoff value of expression: 11.89; *P*-value: 0.009; HR: 1.4, 95% CI: 1.08–1.76), and *RELA* (cutoff value of expression: 12.19; *P*-value <0.001; HR: 1.7, 95% CI: 1.27–2.28) (Figure 7C–F).

**Figure 8:**
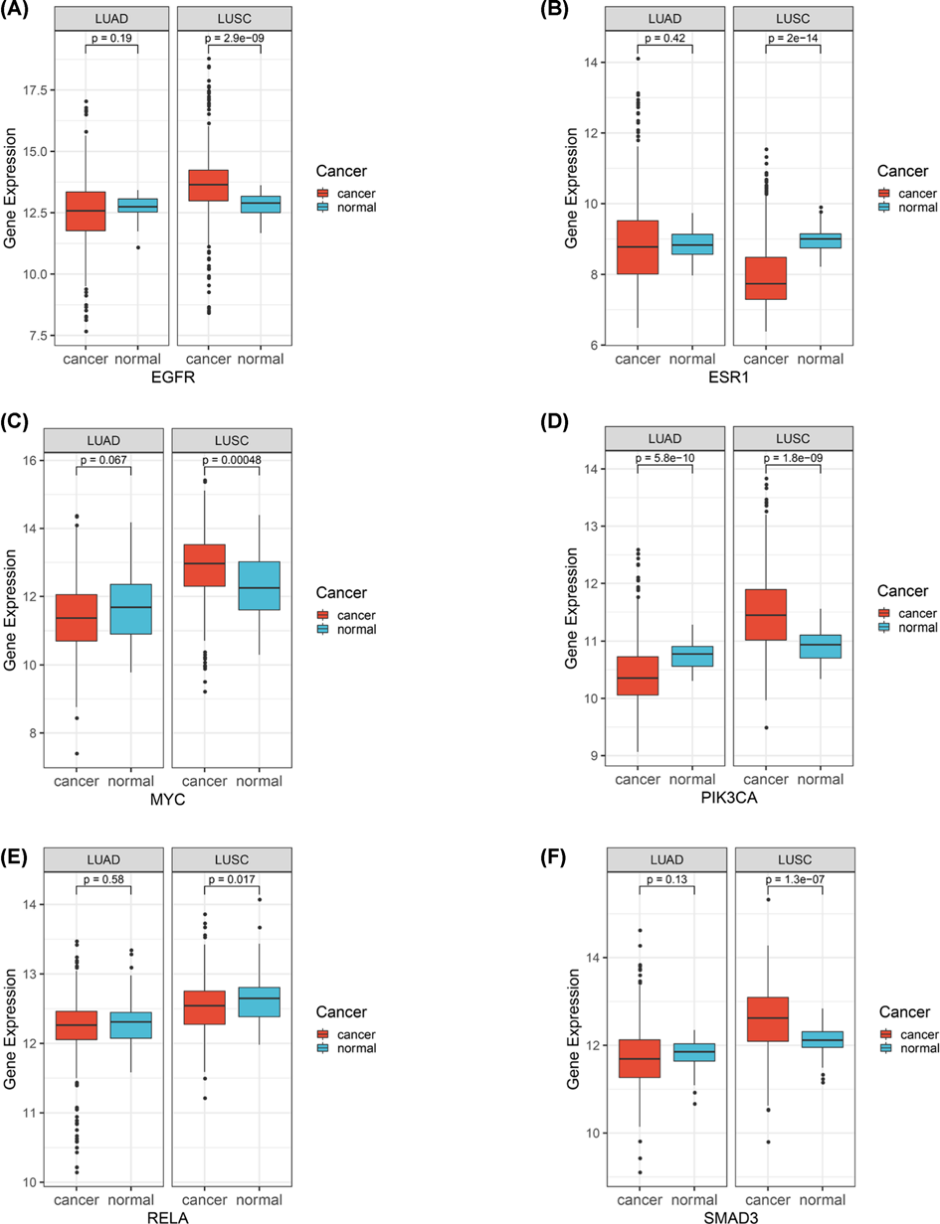
Expression pattern of *EGFR, ESR1, MYC, PIK3CA, RELA*, and *SMAD3* (**A**–**F**) Expression pattern of *EGFR, ESR1, MYC, PIK3CA, RELA*, and *SMAD3* in NSCLC patients.

Since the gene expression data from TCGA were found to not meet with normal distribution by the Shapiro–Wilk test, the expression difference between the cancer group and the para-carcinoma group was identified by the Wilcoxon's test. Consequently, *EGFR* was lowly expressed in the cancer group versus the normal group in LUAD without a statistical difference (*P*-value: 0.19) (Figure 8A) and this expression pattern was similar to *ESR1* (*P*-value: 0.42), MYC (*P*-value: 0.067), *RELA* (*P*-value: 0.058) and *SMAD3* (*P*-value: 0.13) (Figure 8B,C,E,F).

Besides, as for LUSC, the expression level of *EGFR* was significantly higher in the cancer group compared with the normal group (*P*-value <0.001) (Figure 8A) and this expression pattern was also a resemblance to both *MYC* (*P*-value <0.001) and *SMAD3* (*P*-value <0.001) (Figure 8C,F) compared with the normal group, the expression of *PIK3CA* was significantly lower in LUAD group (*P*-value <0.001) but significantly higher in the LUSC group (*P*-value <0.001) (Figure 8D); both *ESR1* and *RELA* were expressed in a significantly lower level in LUSC group than normal group (*P*-value <0.001; *P*-value <0.017) (Figure 8B,E).

In conclusion, based on the validation of TCGA data, the high expression levels of *EGFR, ESR1, MYC, PIK3CA, SAMD3*, and *RELA* were all significantly in association with the poor survival of NSCLC patients. Additionally, hub target genes of ADI: *EGFR, MYC, PIK3CA*, and *SMAD3* all have a significantly higher expression level in the LUSC group versus the normal group, while both *PIK3CA* and *RELA* had a significantly lower expression level in the LUAD group and LUSC group compared with normal group, respectively. These findings would offer fundamental information regarding the expression pattern of these major hub genes and their impact on NSCLC patients’ survival to further relevant researches.

### Analysis of drugs–compounds–hub target genes–pathways network

**Figure 9:**
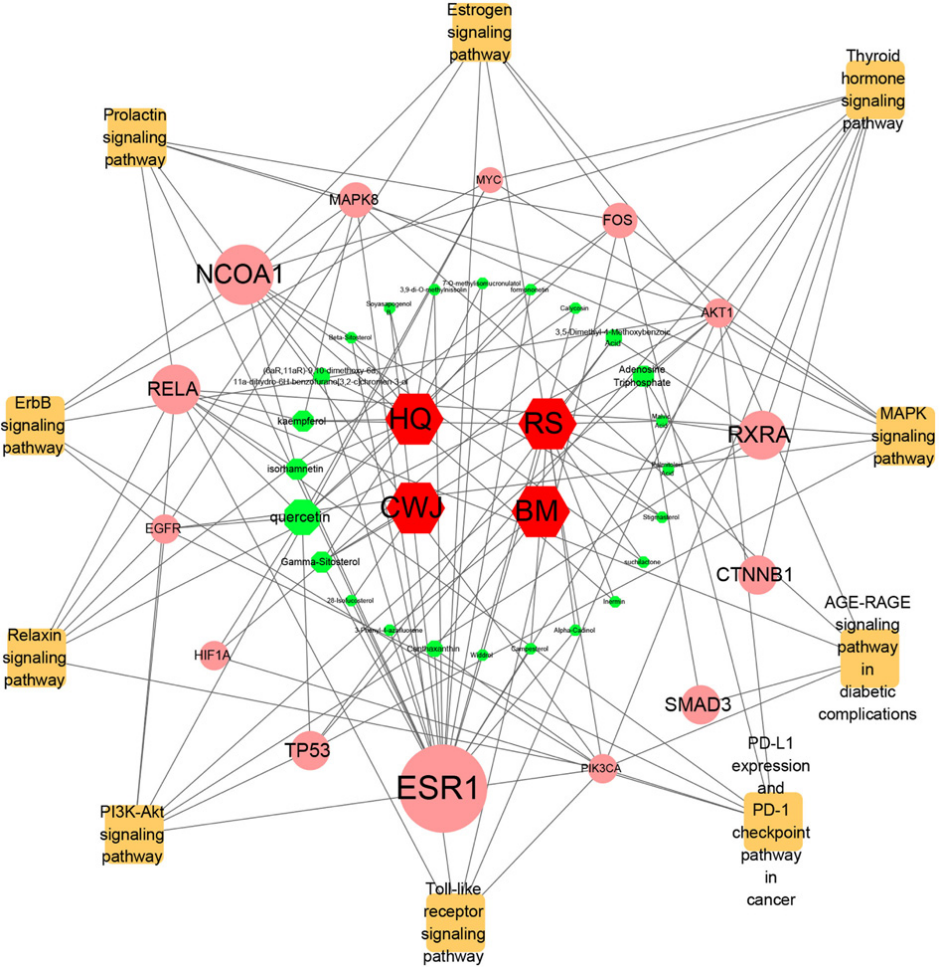
The interaction network of drugs–compounds–hub target genes–pathways The red hexagon nodes stand for drugs of ADI and the cyan octagon nodes stand for compounds. The pink circular nodes represent the hub genes and the orange square nodes represent the KEGG pathways. The greater degree of the hub genes and compounds are, the greater size of the nodes are.

The drugs–compounds–hub target genes–pathways network was utilized to filter the major hub genes and key compounds of ADI to treat lung cancer. This network contained 52 nodes and 126 edges (Figure 9). The size of compound nodes and target gene nodes were positively correlated with their degree in the network. In this network, the hub gene node with the top three value was regarded as a major hub gene, at which key compounds targets to treat lung cancer. Hub genes with the top three degree value in the network included *ESR1* (degree: 15), *NCOA1* (degree: 5), *RXRA* (degree: 3), and *RELA* (degree: 3). In addition, in this network, the compounds that had the top five degree value were considered as key compounds of ADI. They were quercetin, adenosine triphosphate, kaempferol, isorhamnetin, and γ-sitosterol. Their detailed information i presented in Table 2.

**Table 2:** Key compounds of ADI for lung cancer

Drug source	Compound	Molecular structural formula	CAS code
HQ	Quercetin	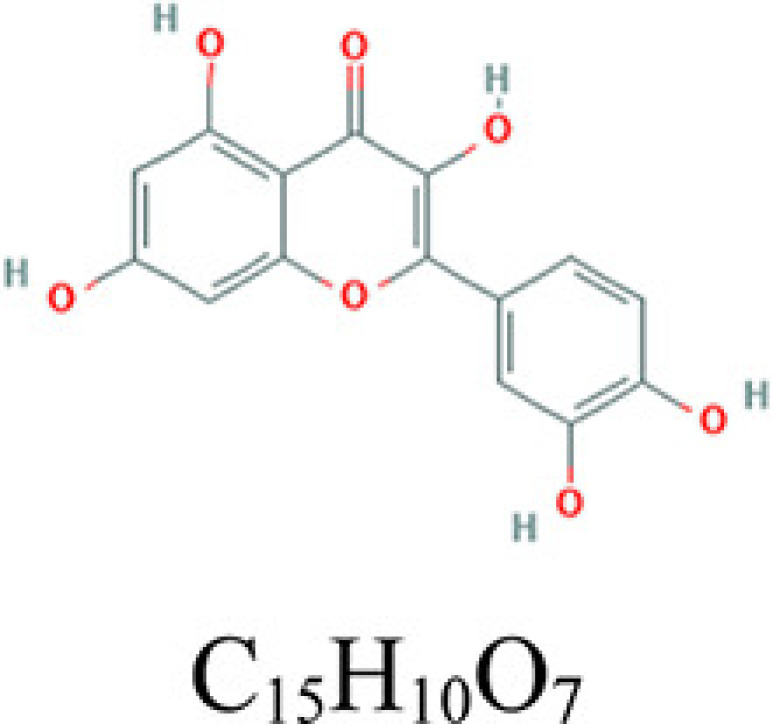	117-39-5
RS	Adenosine triphosphate	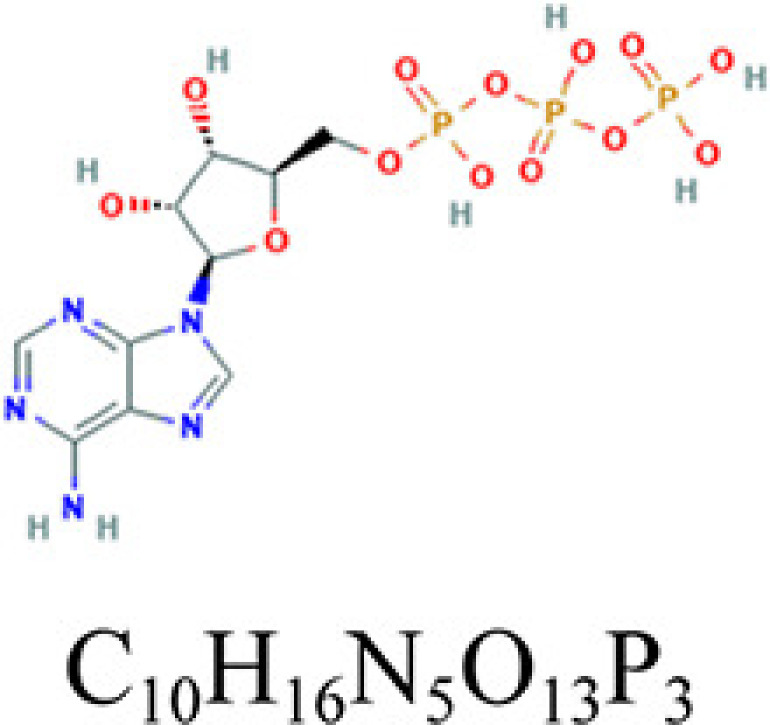	56-65-5
RS HQ	Kaempferol	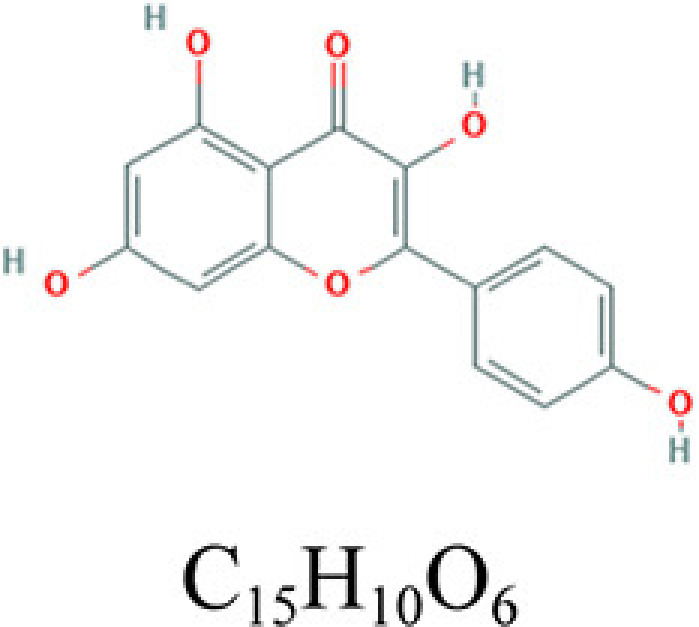	520-18-3
HQ	Isorhamnetin	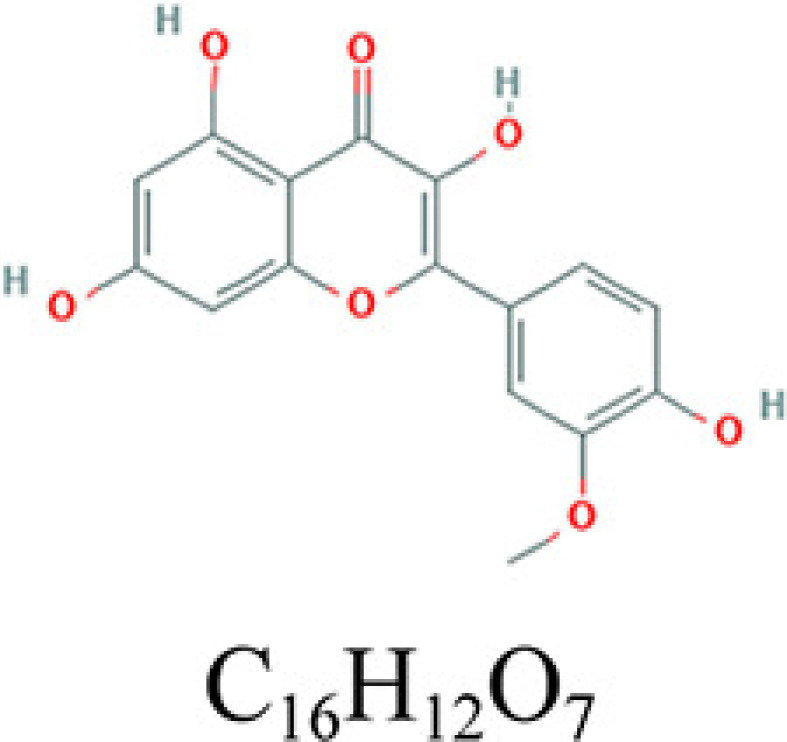	480-19-3
RS HQ CWJ	γ-Sitosterol	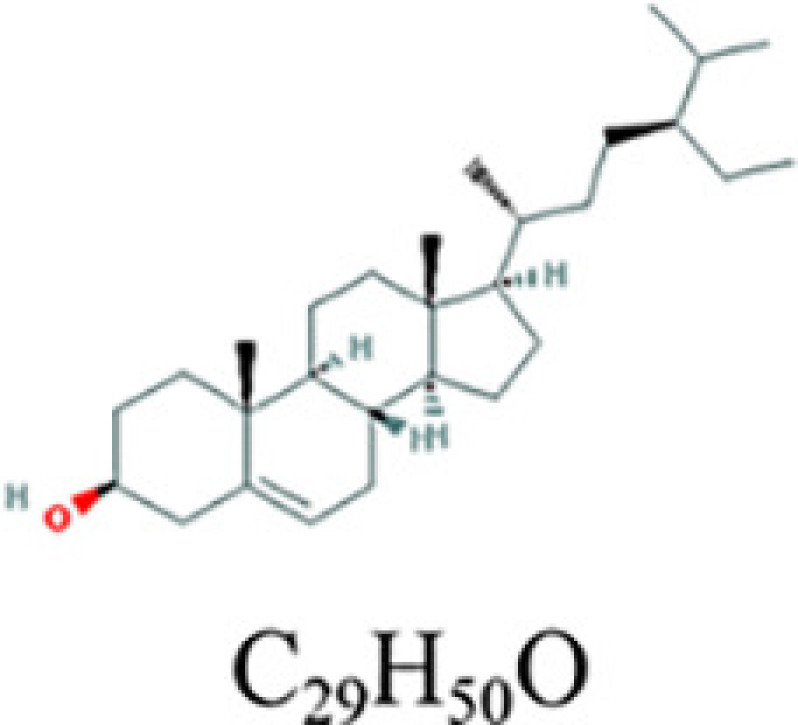	83-47-6

### Outcomes of the molecular docking validation

**Figure 10:**
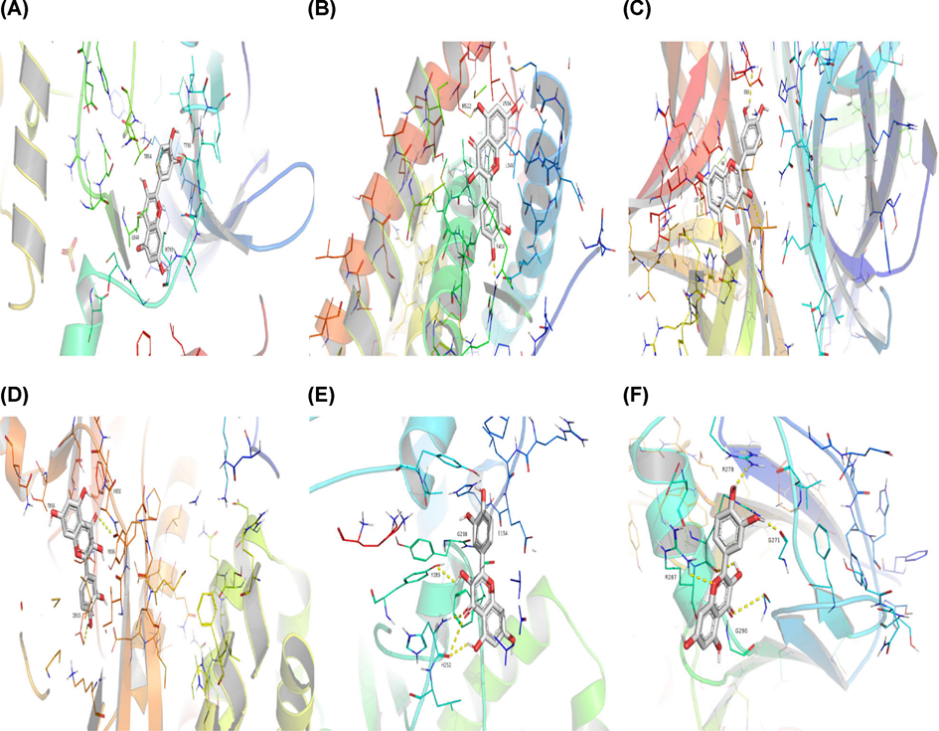
The structures of Quercetin and hub target proteins (**A**) Quercetin with *EGFR*, (**B**) Quercetin with *ESR1*, (**C**) Quercetin with *MYC*, (**D**) Quercetin with *PI3KCA*, (**E**) Quercetin with *RELA*, (**F**) Quercetin with *SMAD3*.

**Figure 11:**
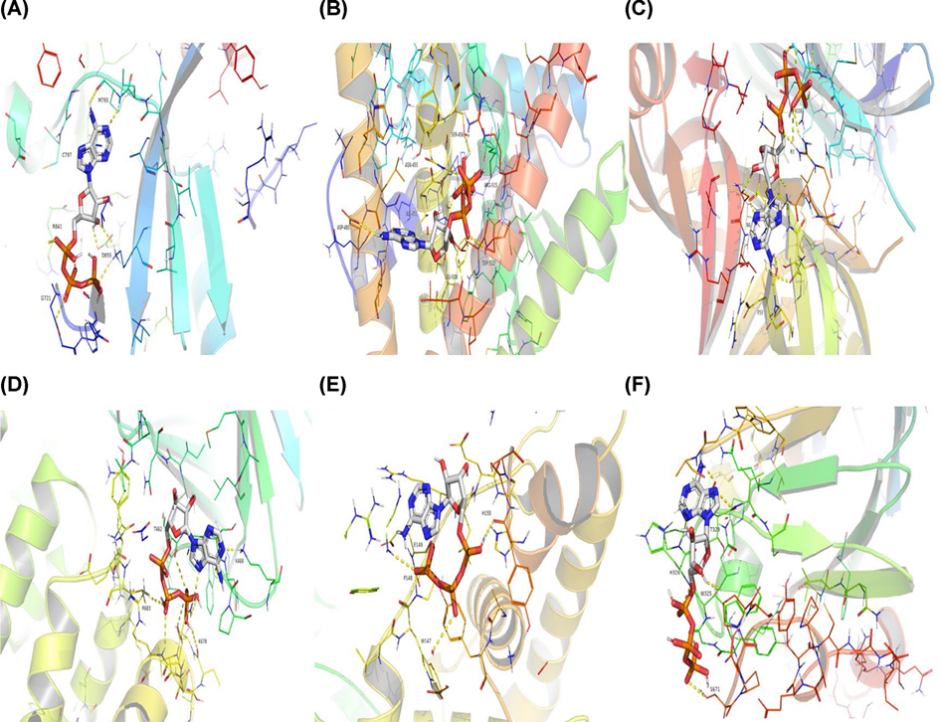
The conformations of Adenosine Triphosphate and hub target proteins (**A**) Adenosine triphosphate with *EGFR*, (**B**) Adenosine triphosphate with *ESR1*, (**C**) Adenosine triphosphate with *MYC*, (**D**) Adenosine triphosphate with *PI3KCA*, (**E**) Adenosine triphosphate with *RELA*, (**F**) Adenosine triphosphate with *SMAD3*.

**Figure 12:**
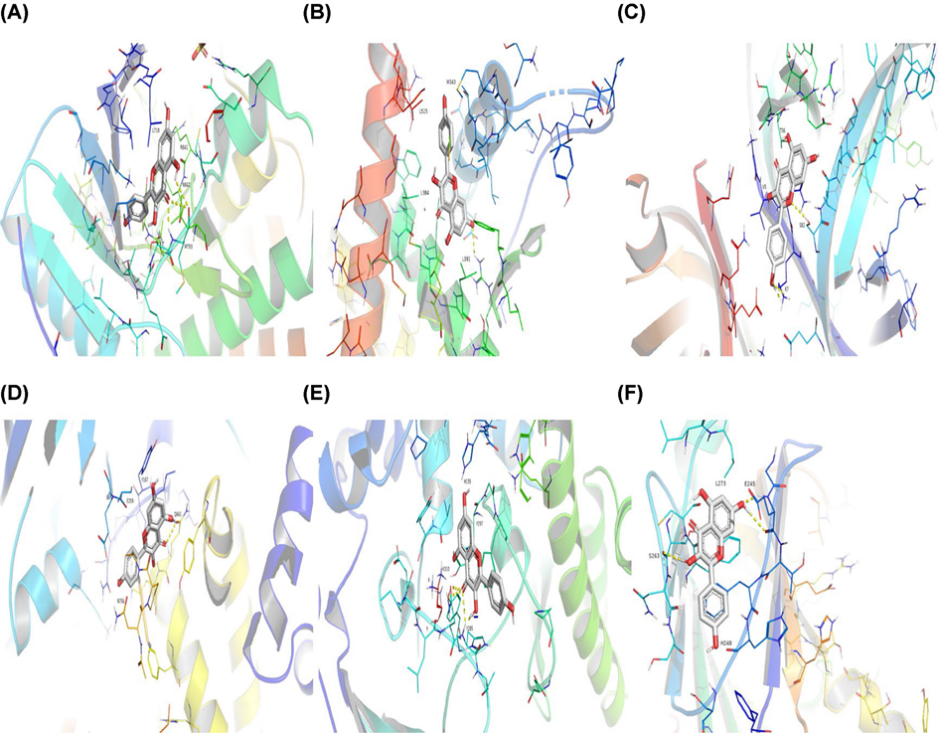
The conformations of Kaempferol and hub target proteins (**A**) Kaempferol with *EGFR*, (**B**) Kaempferol with *ESR1*, (**C**) Kaempferol with *MYC*, (**D**) Kaempferol with *PI3KCA*, (**E**) Kaempferol with *RELA*, (**F**) Kaempferol with *SMAD3*.

**Figure 13:**
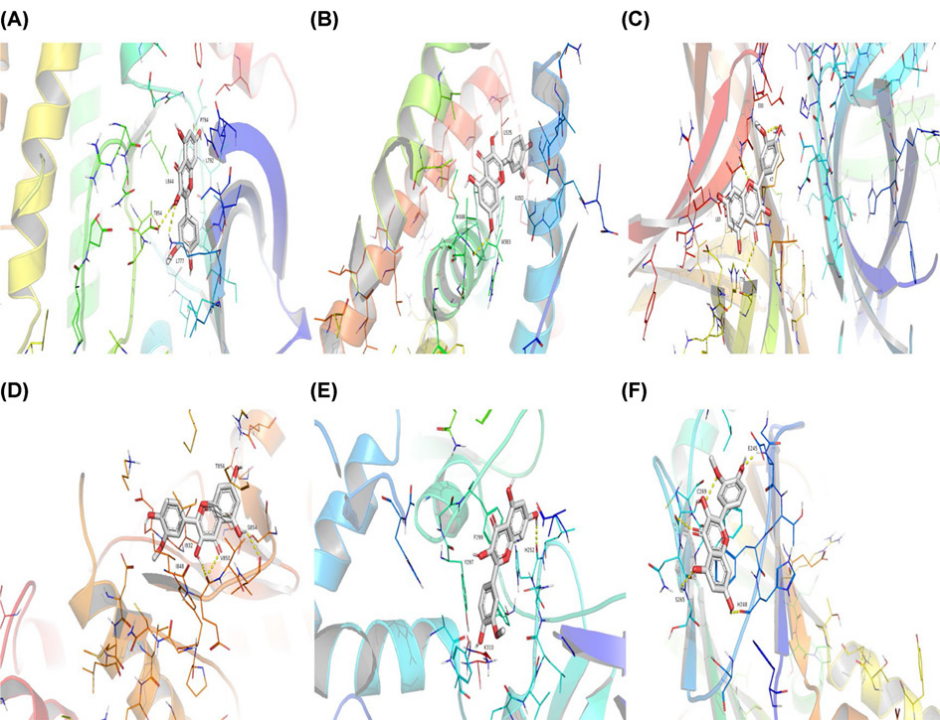
The conformations of Isorhamnetin and hub target proteins (**A**) Isorhamnetin with *EGFR*, (**B**) Isorhamnetin with *ESR1* (**C**) Isorhamnetin with *MYC*, (**D**) Isorhamnetin with *PI3KCA*, (**E**) Isorhamnetin with *RELA*, (**F**) Isorhamnetin with*SMAD3*.

**Figure 14:**
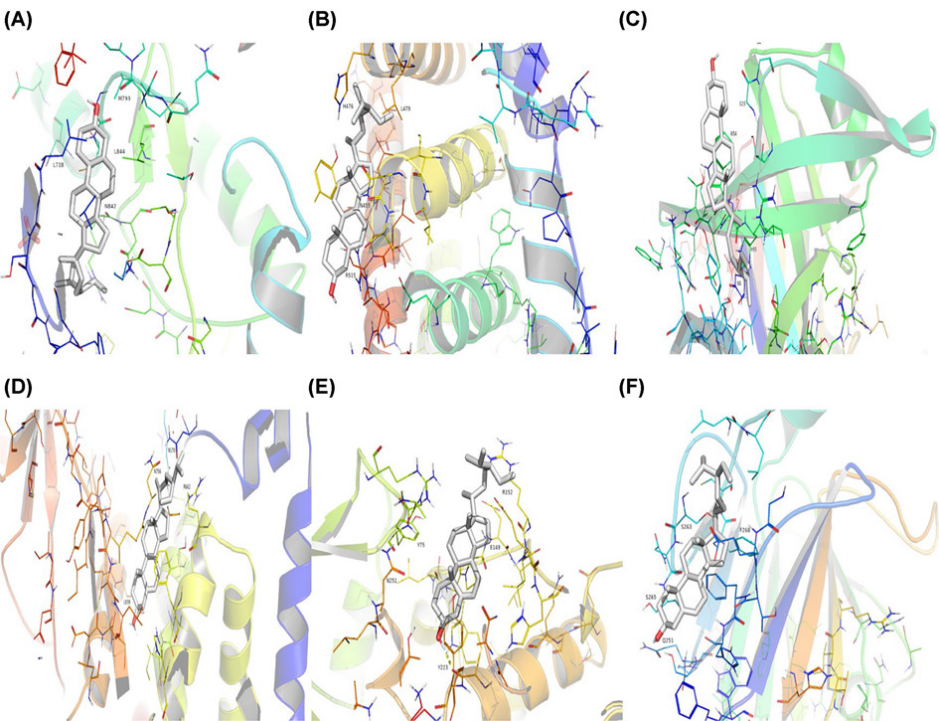
The conformations of γ-Sitosterol and hub target proteins (**A**) γ-Sitosterol with *EGFR*, (**B**) γ-Sitosterol with *ESR1*, (**C**) γ-Sitosterol with *MYC*, (**D**) γ-Sitosterol with *PI3KCA*, (**E**) γ-Sitosterol with *RELA*, (**F**) γ-Sitosterol with *SMAD3*.

After the identification of the key compounds and the hub target genes, the molecular docking between the key compounds and target protein expressed by the hub genes that had impact on NSCLC patients’ survival was executed. The docking affinity score was computed via Autodock Vina and the results were displayed in Table 3. The docking structures of quercetin and the hub target proteins are provided in Figure 10 and the other docking structures of compounds and hub target proteins were shown in the Appendices (Figures 11-14). The average docking affinity score between quercetin, kaempferol, isorhamnetin, γ-sitosterol, and adenosine triphosphate was −8.68, −8.50, −8.42, −7.98, and −7.57, respectively. That is, these key compounds of ADI had good binding interactions between the target proteins expressed by the hub genes that correlated with the survival of NSCLC patients.

**Table 3:** Molecular docking results of the key compounds and hub target protein

Key compound	Hub target protein					
	Docking affinity score (kcal/mol)					
	ESR1	EGFR	PI3KCA	RELA	SAMD3	MYC
Adenosine triphosphate	−7.2	−7.5	−7.6	−7.9	−7	−8.2
γ-sitosterol	−7.2	−8.3	−9	−7.9	−7.5	−8
Isorhamnetin	−7.4	−8.7	−9.1	−8.8	−7.4	−9.1
Kaempferol	−8.4	−8.3	−8.9	−9.3	−7.6	−8.5
Quercetin	−9.1	−8.8	−8.9	−8.9	−7.1	−9.3

## Discussion

Lung cancer is the leading cause of worldwide cancer deaths, in 2017, among men aged 40 years and older and women aged 60 years and older [1]. Consisting of multiple components, TCM formulas produce therapeutic effects through multiple targets, pathways, and BPs. With the effects of improving QoL as well as reducing adverse events caused by chemotherapy, ADI has been widely used for treating lung cancer over the past decade [21]; however, the potential action mechanisms remain unclear. The present study built up associations among the active components, targets, pathways, BPs, and diseases to investigate the underlying mechanisms of ADI for lung cancer.

The drugs–compounds–hub target gene–pathways network exhibited that 114 active compounds in ADI involving 14 hub targets and 10 most relevant pathways were identified. Among the 114 active compounds, quercetin, adenosine triphosphate, kaempferol, isorhamnetin, and γ-sitosterol were the most critical compounds with the high value of degrees in the network. The molecular docking results showed that these key compounds of ADI had good binding interactions withthe target proteins associated with the survival of NSCLC patients (Table 3; Figure 10 and Appendices: Figures 11-14). Quercetin, a flavonoid with potential chemopreventive properties, is reported to possess the effects of anticancer, anti-inflammatory, and anti-proliferation on many types of cancer including lung cancer [43,44]. Adenosine triphosphate is an important substance of cancer metabolism. The elevated adenosine triphosphate in the extracellular environment participates in immune responses and inflammation and induces anticancer effects [45,46]. The drop of adenosine triphosphate levels in lung cancer cell lines is delayed by curcumin, an active herb ingredient, which can promote cell apoptosis process and exert anti-carcinogenic and anti-tumor effects [47]. As for kaempferol, it is widely recognized to exert anticancer effects against lung cancer by inducing cell cycle arrest and apoptosis as well as suppressing tumor growth [48,49]. Previous experimental research has shown that kaempferol can enhance the radiosensitivity of lung cancer cells through inhibiting AKT/PI3K and ERK pathways and activating the mitochondria apoptosis pathway [50]. Isorhamnetin is a natural monomer with antitumor effects on lung cancer cell lines. A recent study finds that isorhamnetin is associated with anti-proliferation effects and cell apoptosis induction, and it could inhibit cancer cell growth and alters the expression of apoptosis-related genes involving *Bcl-2, Bax, Caspase-3*, and so on [51]. In addition, isorhamnetin increases protein levels of light chain 3-II when autophagy is initiated, up-regulates the expression of Beclin1, an activator of PI3K, and promotes accumulation of monodansylcadaverine, an *in vivo* marker for autophagic vacuoles, which confirm the effects of isorhamnetin on autophagy induction in NSCLC cells [52]. As for γ-sitosterol, a previous study shows that it can induce cell apoptosis in lung cancer cell lines (A549) by G_2_/M cell cycle arrest, leading to growth inhibitory effects on lung cancer cells [53]. In the present study, quercetin, adenosine triphosphate, kaempferol, isorhamnetin, and γ-sitosterol were considered important compounds of ADI for lung cancer treatment, which may become the future research directions.

Our results from the PPI network highlighted 14 hub genes, of which *ESR1, NCOA1, RXRA*, and *RELA* were identified as major hub genes in the drugs–compounds–hub target genes–pathways network. *ESR1* is a transcription factor with the function of growth suppression [54]. High expression of *ESR1* is regarded as an independent prognostic factor related to metastasis NSCLC, which is conducive to divide NSCLC patients into various prognosis groups, guiding the administration of chemotherapy [55]. As to *RELA*, research results from Giopanou et al. [56] indicate that *RELA* is a protein-coding gene highly expressed in NSCLC cells, and also increased in tumors with higher degrees of inflammation, thus tumor-associated inflammation can be predicted. *RELA* is one of the transcription factors mediated inflammatory response and involved in cancer development [57]. With regard to *NCOA1*, Qin et al. have demonstrated that the overexpression of *NCOA1* could promote the breast cancer cells to disseminate into blood circulation and accelerate the lung metastasis [58], while the associations between *NCOA1* and lung cancer cells have not been fully studied and deserve further exploration. *RXRA* is an oncogene involved in different types of cancer [59]. A recent study indicates that *RXRA* is involved in cell cycle regulation and lung cancer development, in this case, it is regarded as the potential target gene of lung cancer [60]. Accordingly, we speculated that the mechanisms of ADI on treating lung cancer were associated with the regulation of *ESR1, NCOA1, RXRA*, and *RELA*.

In the present study, the survival analysis revealed that the major hubs including *ESR1* and *RELA* were verified prognostic value on the overall survival of lung cancer. Results from previous studies provided some supports for our findings. According to Brueckl et al., the adjuvant treatment is associated with a reduced death risk in NSCLC patients with low *ESR1* expression, but the same response did not appear in the patients with high *ESR1* expression [61]. Similarly, the higher expression level of *RELA* was proved to associate with the poor survival of patients with lung cancer [62]. In addition, a clinical trial includes 82 NSCLC patients who have received chemotherapy regimen previously or are considered not applicable for chemotherapy, indicating that high *PIK3CA* gain combined with high phosphatase and tensin homolog (*PTEN*) loss have shorter median overall survival compared with low *PIK3CA* gain and *PTEN* loss (4.93 vs 12.3 months, *P*-value <0.001) [63]. Lin et al. have reported that *SMAD3* genetic variation rs4776342 is associated with poorer overall survival (HR = 1.25, 95% CI: 1.06–1.47, *P*-value <0.01), and multiple wild-type *SMAD3* genotypes benefited the survival in both the chemo-radiation treatment group as well as the chemotherapy treatment group [64]. Kim et al. recruited a total of 84 NSCLC patients to evaluate the importance of *MYC*, and their study showed that patients with expression of both *MYC* and *PD-L1* had a poorer disease-free survival (7.1 vs 31.1 months, *P*-value: 0.011) than patients without double-positive expression pattern [65]. Besides, a retrospective cohort analysis including 285 lung cancer patients reveals that the *EGFR* mutations group has a better median overall survival of 20.0 months than that of 11.0 months in the non-mutated group (*P*-value: 0.007) [66]. Therefore, all of these six hub genes might have significant impacts on the survival of lung cancer patients and ADI might benefit patients’ survival by regulating them. Interestingly, a group of sex determining region Y (SRY)-related high-mobility group (HMG) box (SOX) proteins (SOX1, SOX2, SOX4, SOX3, SOX5, SOX7, ect.) was identified as the putative target gene of lung cancer in our study (Appendix C, Supplementary material). Recently, the SOX family is regarded to have the important transcription factors associated with the proliferation, migration and invasion, and metastasis of cancer [67]. For example, it is proved that SOX7 is a suppressive target gene expressed in the NSCLC cells and the forced expression of SOX7 in NSCLC cell lines could markedly reduce the cancer cell growth and enhanced their apoptosis [68]. The interaction and relation of hub target genes of ADI and the gene from SOX family deserve further research.

As revealed from the GO enrichment analysis, ADI was highly associated with gland development and cellular response to oxidative stress. As for gland development, a previous research [69] illustrates that gland development can be regulated by the type 1 insulin-like growth factor receptor which is overexpressed in lung cancer cells and mediated cell proliferation and metastasis. Furthermore, oxidative stress is reported to exert important roles in the occurrence, development, and progression of carcinogenesis by participating in multiple signaling pathways, inflammatory response, and cell apoptosis [70]. Cigarette smoke appeared to be the main cause of lung cancer, can generate oxidative stress and trigger airway inflammation, contributing to high levels of reactive oxygen species (ROS) and activation of the MAPK pathway [71]. Hence, ADI exerted therapeutic effects on lung cancer probably concerning the above BPs.

The findings of the KEGG pathway annotation indicated that ADI might exert therapeutic effects mainly by regulating the thyroid hormone signaling pathway, MAPK signaling pathway, and PI3K-Akt signaling pathway. The thyroid hormone regulates cellular activities, such as tissue differentiation, cell growth, and metabolism. From the previous experimental research [72], the thyroid hormone, initiated by integrin αvβ3, stimulates the proliferating cell nuclear antigen accumulation in NSCLC cells, supporting its effects on cell proliferation. *ESR1* can bind to estrogens to regulate thyroid cell proliferation and cell survival [73]. The thyroid hormone produces effects depending on the thyroid hormone receptors which exert transcription function by binding the regulatory proteins [74]. Moreover, the thyroid hormone is also reported to promote tumor proliferation by activating the PI3K, the ERK1/2, and MAPK pathways [75,76]. The MAPK signaling pathway is involved in the progression and prognosis of NSCLC patients by regulating cell proliferation, differentiation, and apoptosis [77]. According to Wang et al., the MAPK signaling pathway is regulated by Circ-ZKSCAN1, a circular RNA of great importance in carcinogenesis, to advance the progression of NSCLC [78]. As for the PI3K-Akt signaling pathway, it is proved to associate with multifarious cellular functions and apoptosis in NSCLC cells [79]. A previous study has shown that the hub gene *RXRA* can influence cell differentiation, cell migration, and provoke tumor suppression via the PI3K-Akt signaling pathway [80]. The major hub gene *RELA* produces autophagy inhibitory effects in cancer cells where the PI3K-Akt signaling pathway is activated and promotes tumor invasion, concluding that autophagy inhibition may be a therapeutic strategy for treating cancers [81]. Therefore, the active compounds of ADI may act on these signaling pathways in treating lung cancer.

In this research, we identified the key compounds, hub gene targets, relevant pathways of ADI by comprehensive NP strategies and molecular docking. In addition, the expression patterns of the hub target genes as well as their impacts on NSCLC were explored by the TCGA database (1042 NSCLC patients). Furthermore, the interactions between the key compounds and the hub target genes were validated by the molecular docking technique. However, there were some limitations in our study. On one hand, our study predicted and verified the molecular mechanisms of ADI on lung cancer at a system level, while there was no sufficient experimental evidence to validate these results right now. Though our findings provided some interesting evidence for the further study of ADI, potential associations among compounds, target genes, and pathways still require further experimental confirmations. On the other hand, analysis of TCGA data implied that six hub genes of ADI possessed impact on the survival of NSCLC patients, however, more researches are essential to further identify whether ADI could benefit NSCLC patients' survival via regulating these hub genes. In summary, the present study has demonstrated that the potential mechanisms of ADI for treating lung cancer involve multiple active compounds, target genes, and the signaling pathways, providing references for clinical application of ADI and future researches.

## Conclusion

Though ADI has been applied for lung cancer for a few decades, pharmacological mechanisms of ADI remain unclear. Based on the NP approach, the active compounds of ADI, including quercetin, adenosine triphosphate, kaempferol, isorhamnetin, and γ-sitosterol, were screened out. *ESR1, NCOA1, RXRA*, and *RELA* were identified as major hub genes. The survival analysis revealed that ESR1 and *RELA* together with the hub genes including *EGFR, PIK3CA, MYC*, and *SMAD3* were proved to have prognostic value on NSCLC patients’ survival. These target genes also had great docking energy with the key compounds of ADI. ADI may produce curative effects on lung cancer by regulating the thyroid hormone signaling pathway, MAPK signaling pathway, and PI3K-Akt signaling pathway. The findings of the present study indicated that the pharmacological mechanisms of ADI for lung cancer involved diverse compounds, targets, BPs, and signaling pathways, and provided references for further researches.

## Supplementary Material

Supplementary material Appendix A-D

## Data Availability

The data used to support the findings of the present study are available from the corresponding author upon request.

## Abbreviations

ADI: Aidi injection
Batman-TCM: Bioinformatics Analysis Tool for Molecular mechANism of Traditional Chinese Medicine
BM: Banmao
BP: biological process
CC: cellular component
CI: confidence interval
CWJ: Ciwujia
GO: Gene Ontology
HQ: Huangqi
HR: hazard ratio
KEGG: Kyoto Encyclopedia of Genes and Genomes
LUAD: lung adenocarcinoma
LUSC: lung squamous cell cancer
MF: molecular function
NP: network pharmacology
NSCLC: non-small cell lung cancer
OB: oral bioavailability
OMIM: Online Mendelian Inheritance in Man
PPI: protein–protein interaction
QoL: quality of life
RS: Renshen
SOX: sex determining region Y-related high-mobility group box
STRING: The Search Tool for the Retrieval of Interacting Genes
TCGA: The Cancer Genome Atlas
TCM: Traditional Chinese medicine
TCMP: TCM preparation
TCMSP: Traditional Chinese Medicine Systems Pharmacology database
TTD: Therapeutic Target Database
