# Supplementary material for: Exploring the mechanism of aidi injection for lung cancer by network pharmacology approach and molecular docking validation
Source: Biosci Rep. 2021 Feb 12;41(2):BSR20204062. doi: 10.1042/BSR20204062 (PMC7881165; doi:10.1042/BSR20204062)
Supplement: Supplementary material Appendix A-D [file BSR-2020-4062_supp.pdf]

Appendices

Appendix A. Supplementary material: Drugs and compounds of ADI

Appendix B. Supplementary material: Compounds and putative target genes of ADI

Appendix C. Supplementary material: Putative target genes of lung cancer from Genecard, OMIM and TTD database

Appendix D. Supplementary material: Common putative target genes information between ADI and lung cancer
